# Quality of life in refractory Graves’ hyperthyroidism: a cross-sectional study comparing long-term ATDs therapy versus L-T4 replacement after iodine-131

**DOI:** 10.3389/fendo.2026.1776734

**Published:** 2026-03-23

**Authors:** Anqi Yuan, Zhe Yan, Jialu Wu, Yifei Song, Hui Huang

**Affiliations:** Department of Endocrinology and Metabolism, West China Hospital, Sichuan University, Chengdu, China

**Keywords:** antithyroid therapy, Graves’ disease, iodine-131 therapy, patients-reported outcomes, quality of life, ThyPRO-39

## Abstract

**Objective:**

Evaluate and compare the patient-reported quality of life (QoL) outcomes in Graves’ disease patients on long-term antithyroid drugs (ATDs) therapy and levothyroxine (L-T4) replacement after iodine-131, and explore factors influencing QoL.

**Methods:**

This single-center cross-sectional observational study included refractory Graves’ hyperthyroidism patients who were followed in outpatient between January 2015 and July 2024. Patients were divided into two groups based on their current treatment: the ATDs group and the L-T4 group. We collected demographic and clinical characteristics and assessed cross-sectionally QoL using the thyroid-related patient-reported outcome-39 questionnaire (ThyPRO-39). We compared QoL scores between the two groups and examined factors associated with QoL using regression analyses.

**Results:**

A total of 499 patients were enrolled, of whom 300 completed the ThyPRO-39 (response rate 60.1%; 172 in the ATDs group and 128 in the L-T4 group). Compared with the ATDs group, patients in the L-T4 group had significantly higher scores across several subscales, including hyperthyroid symptoms, hypothyroid symptoms, eye symptoms, anxiety, emotional susceptibility, social life, daily life, appearance, overall QoL, and the composite scale (all P < 0.05), indicating lower QoL. In the multiple linear regression analysis, female was positively correlated with the scores of hypothyroidism symptoms, tiredness and depressivity (all P < 0.05). Male was negatively correlated with the score of the composite scale, while L-T4 group was positively correlated with the score of the composite scale (all P < 0.05).

**Conclusion:**

In patients with refractory Graves’ hyperthyroidism, female patients and those undergoing long-term L-T4 replacement therapy after iodine-131 treatment were associated with lower QoL.

## 1 Introduction

Graves’ disease (GD) is the most common cause of hyperthyroidism in iodine-sufficient regions, accounting for 70–80% of all hyperthyroid cases. It occurs mostly in women aged 20–50 years, about 3% of women and 0.5% of men are affected (1–3). The primary treatment modalities for GD include antithyroid drugs (ATDs) therapy, radioactive iodine-131 (RAI) therapy, and thyroidectomy. Each approach presents distinct advantages and limitations. Given the invasive nature of surgery and the risk of perioperative complications, thyroidectomy is seldom selected as the first-line treatment (4). Currently, ATDs are the most commonly used treatments for initial onset of Graves’ hyperthyroidism, with varying proportions across different countries and regions (5, 6). In the Asia-Pacific and Europe regions, 70.6–85.7% of patients opt for ATDs therapy (7, 8). In North America, approximately 40.5% of patients choose ATDs, with a growing trend in recent years according to recent literature report (9). ATDs therapy offers several advantages in the management of Graves’ hyperthyroidism, including ease of administration and the absence of permanent hypothyroidism. For Graves’ hyperthyroidism, the standard treatment duration is 12–18 months, with discontinuation recommended after normal thyroid function and negative TRAb levels are sustained (10). Nevertheless, the incidence of relapse post-discontinuation is high, ranging from 50% to 70%, thus limiting the utilization of ATDs (11–13). While iodine-131 therapy has the advantages of high remission rates and ease of administration, it carries a higher risk of hypothyroidism and Graves’ orbitopathy, raising concerns among both physicians and patients. However, hypothyroidism following iodine-131 therapy seems inevitable and reflects effective hyperthyroid treatment, necessitating lifelong thyroid hormone replacement to maintain stable thyroid function.

Recent studies indicate that long-term, low-dose ATDs therapy is safe, well-tolerated, and effective in maintaining thyroid function, reducing relapse rates, and improving remission rate (12, 14–17). Considerable debate has arisen regarding whether patients with hyperthyroidism who are prone to relapse during ATDs therapy should continue with long-term, low-dose ATDs treatment or undergo iodine-131 therapy followed by long-term levothyroxine (L-T4) replacement. However, the evaluation of treatment outcomes tends to focus on objective clinical markers, often neglecting patients’ subjective experiences. With the increasing incidence of Graves’ disease and relatively low mortality rates, relying solely on recurrence and remission rates to assess clinical outcomes has limitations. Patient-reported outcomes, including quality of life (QoL), are crucial for evaluating the overall treatment effect (18). Although treatment can normalize thyroid function in GD patients, many still face psychological, emotional, and social challenges, impacting their QoL (18–20). Research on QoL in Graves’ disease remains limited, with inconsistent conclusions in existing studies. Given that both therapeutic approaches necessitate prolonged medication use, it is crucial to evaluate and compare their respective effects on patients’ QoL. Therefore, in this study, we enrolled patients with refractory Graves’ hyperthyroidism who had received either long-term ATDs therapy or iodine-131 therapy followed by long-term L-T4 replacement. Participants were stratified into two groups (ATDs group vs. L-T4 group) based on their treatment approach. QoL was assessed to compare the impact of these two therapeutic strategies on patient-reported outcomes, with the ultimate goal of generating more patient-centered data to better monitor health status, inform clinical decision-making and optimize long-term management in GD.

## 2 Methods

### 2.1 Study design and population

This cross-sectional study was conducted at West China Hospital of Sichuan University, a large tertiary academic medical center and a major referral center located in Chengdu, Sichuan Province, China. The hospital has approximately authorized 4,900 beds and recorded over 5 million offline outpatient and emergency visits, with >260,000 inpatient discharges annually. The Department of Endocrinology and Metabolism has 68 inpatient beds and provides over 200,000 outpatient visits and about 3,000 inpatient discharges per year. Approximately 30,000 patients with hyperthyroidism are under follow-up each year. The study protocol was reviewed and approved by the Biomedical Ethics Committee of West China Hospital, and written informed consent was obtained from all participants.

The inclusion criteria were as follows (1): age ≥18 years (2); a confirmed diagnosis of Graves’ hyperthyroidism treated with either oral ATDs or iodine-131 therapy followed by levothyroxine replacement for at least 24 months (3); stable thyroid function within the past 6 months, with TSH and free T4 (FT4) levels within the normal range (4); good treatment adherence and ability to complete follow-up assessments. Patients were excluded if they had (1): severe comorbid organic diseases involving the cardiovascular, cerebrovascular, hepatic, pulmonary, renal, hematologic, or endocrine systems (e.g., stroke, heart failure, renal failure, malignancy) (2); diagnosed psychiatric disorders such as anxiety, depression, or bipolar disorder (3); pregnancy or postpartum status (4); cognitive or reading impairments (5); incomplete clinical data or poor compliance with study procedures.

### 2.2 Collection of patient data

Demographic and clinical characteristics were collected for all enrolled patients. QoL was assessed cross-sectionally using the ThyPRO-39 questionnaire at follow-up visits in 2024, whereas baseline demographic, clinical, and laboratory variables were retrospectively extracted from the electronic medical records of patients under follow-up at our center since January 2015. January 2015 was chosen as the starting point because, from that time onward, our outpatient electronic medical record system became more mature and accessible, with more complete data capture and improved suitability for retrospective retrieval. This timeframe also ensured a sufficiently long observation window to identify patients who had been on their current treatment strategy for ≥24 months and had stable thyroid function in the preceding 6 months at the time of questionnaire completion. Follow-up data were collected during the follow-up visit. The interval between follow-up measurements and questionnaire completion was no more than 3 months. The variables included sex, age at disease onset, initial presenting symptoms, disease duration, the presence of Graves’ orbitopathy, and chronic comorbidities (including hypertension, diabetes mellitus, hyperlipidemia, and osteoporosis). Additional clinical variables included history of hyperthyroidism relapse, duration of therapy, reported adverse drug reactions, and reasons for choosing radioactive iodine-131 therapy. Baseline laboratory parameters at the time of diagnosis were recorded, including serum levels of thyroid-stimulating hormone (TSH), free triiodothyronine (FT3), free thyroxine (FT4), thyroglobulin antibody (TgAb), thyroid peroxidase antibody (TPOAb), and TSH receptor antibody (TRAb), as well as thyroid ultrasound findings. During follow-up, the aforementioned biochemical and imaging indicators were remeasured to assess thyroid function and autoimmune status. Further, peripheral thyroid hormone sensitivity was assessed using the FT3/FT4 ratio. Central thyroid hormone sensitivity was evaluated using the TSH index (TSHI) and the thyrotroph thyroxine resistance index (TT4RI). TSHI was calculated as ln [TSH (mIU/L)] + 0.1345 × FT4 (pmol/L), and TT4RI was calculated as FT4 (pmol/L) × TSH (mIU/L) (10).

### 2.3 Laboratory investigations

All laboratory analyses were performed at the Department of Laboratory Medicine, West China Hospital, Sichuan University. Serum levels of TSH, FT3, FT4, TgAb, TPOAb, and TRAb were measured using a Roche electrochemiluminescence immunoassay analyzer (Modular E170D) with corresponding commercial assay kits. The reference ranges for laboratory parameters were as follows: TSH: 0.27–4.2 mIU/L; FT3: 3.60–7.50 pmol/L; FT4: 12.0–22.0 pmol/L; TgAb: <115 IU/mL; TPOAb: <34 IU/mL; TRAb: <1.75 IU/L. When measured values exceeded the upper or lower limits of the assay’s reference range, they were recorded as the respective limit value.

### 2.4 Evaluation of thyroid volume

Experienced sonographers performed thyroid ultrasonography using the LOGIQ 500 ultrasound system (GE Healthcare). Thyroid volume was subsequently estimated based on the ultrasound measurements. The volume of each thyroid lobe was calculated using the standard ellipsoid formula: height (cm) × width (cm) × depth (cm) × 0.52 = lobe volume (mL). The total thyroid volume was obtained by summing the volumes of the left and right lobes.

### 2.5 Measurement of QoL

QoL was assessed using the ThyPRO-39 questionnaire. Previous validation in Chinese patients with benign thyroid diseases demonstrated good content validity (I-CVI 0.830–1.000; S-CVI 0.952), internal consistency (Cronbach’s α = 0.916), split-half reliability (0.711), and test–retest reliability (0.722–0.858 over 2–3 weeks), with criterion validity supported by correlations with SF-36 (21). A traditional Chinese adaptation study also supported convergent and known-group validity in ethnic Chinese patients, although internal consistency for some domains was below the conventional threshold (22). The ThyPRO-39 questionnaire consists of 39 items divided into 12 subscales and one single-item scale evaluating overall QoL. The subscales include goiter symptoms, hyperthyroid symptoms, hypothyroid symptoms, eye symptoms, tiredness, cognitive complaints, anxiety, depressivity, emotional susceptibility, impaired social life, impaired daily life, appearance and overall QoL. The composite scale comprises 22 items from several subscales (tiredness, cognitive complaints, anxiety, depressivity, emotional susceptibility, impaired social life, impaired daily life, appearance and overall QoL). Raw scores for each subscale were converted into standardized scores ranging from 0 to 100 based on a scoring manual and formula, where higher scores indicate poorer QoL (22, 23).

In addition to the validated ThyPRO-39 (13 scales; 39 items), we appended one study-specific, non-validated exploratory item immediately after item 39 to capture participants’ perceived recovery. The new reads: “After taking your medication, do you feel that your QoL has partially or completely returned to its pre-morbid level?” Participants selected one of five options: “No recovery (0%)”, “25% recovery”, “50% recovery”, “75% recovery”, or “Complete recovery (100%)”. This item was analyzed and reported separately and was not included in any validated ThyPRO-39 domain scores or the composite score.

Before completing the questionnaire, all participants received a thorough explanation of the study’ s objectives, procedures, significance, potential risks and benefits, and subsequently provided written informed consent. Participants were assured that their responses would be stored securely by the research team, and that all data would be de-identified prior to analysis to protect personal information and privacy.

### 2.6 Statistical analysis

All analyses were conducted with IBM SPSS Statistics 26.0 (IBM Corp., Armonk, NY, USA). Two-tailed tests were applied throughout, and p < 0.05 was considered statistically significant. The Kolmogorov–Smirnov test was used to assess the normality of continuous variables. Data are reported as mean ± standard deviation (SD) for normally distributed variables and as medians (first quartile (Q1)–third quartile (Q3)) for non-normal distributions. Categorical variables were compared between groups using the Chi-square test. For continuous variables, inter-group differences were examined with the independent-samples t test when normality assumptions were met and with the Mann-Whitney U test or Wilcoxon signed-rank test when they were not. Spearman rank-order correlation was employed to explore associations among variables. The scores of each ThyPRO-39 subscale and the composite score were used as dependent variables. The composite score was the primary outcome. The subscale scores were secondary outcomes. Variables that were both clinically and statistically significant, and that showed no obvious correlation with any other variables, were selected as independent variables. Multiple linear regression analysis was performed using the “input” method. Furthermore, key linear regression assumptions were assessed. Residual normality was evaluated using Q-Q plots; homoscedasticity was examined by residuals-versus-fitted plots; influential outliers were assessed using standardized residuals; and multicollinearity was assessed using variance inflation factors (VIFs). No substantial violations of the assumptions were detected.

## 3 Results

### 3.1 Study population

**Figure 1:**
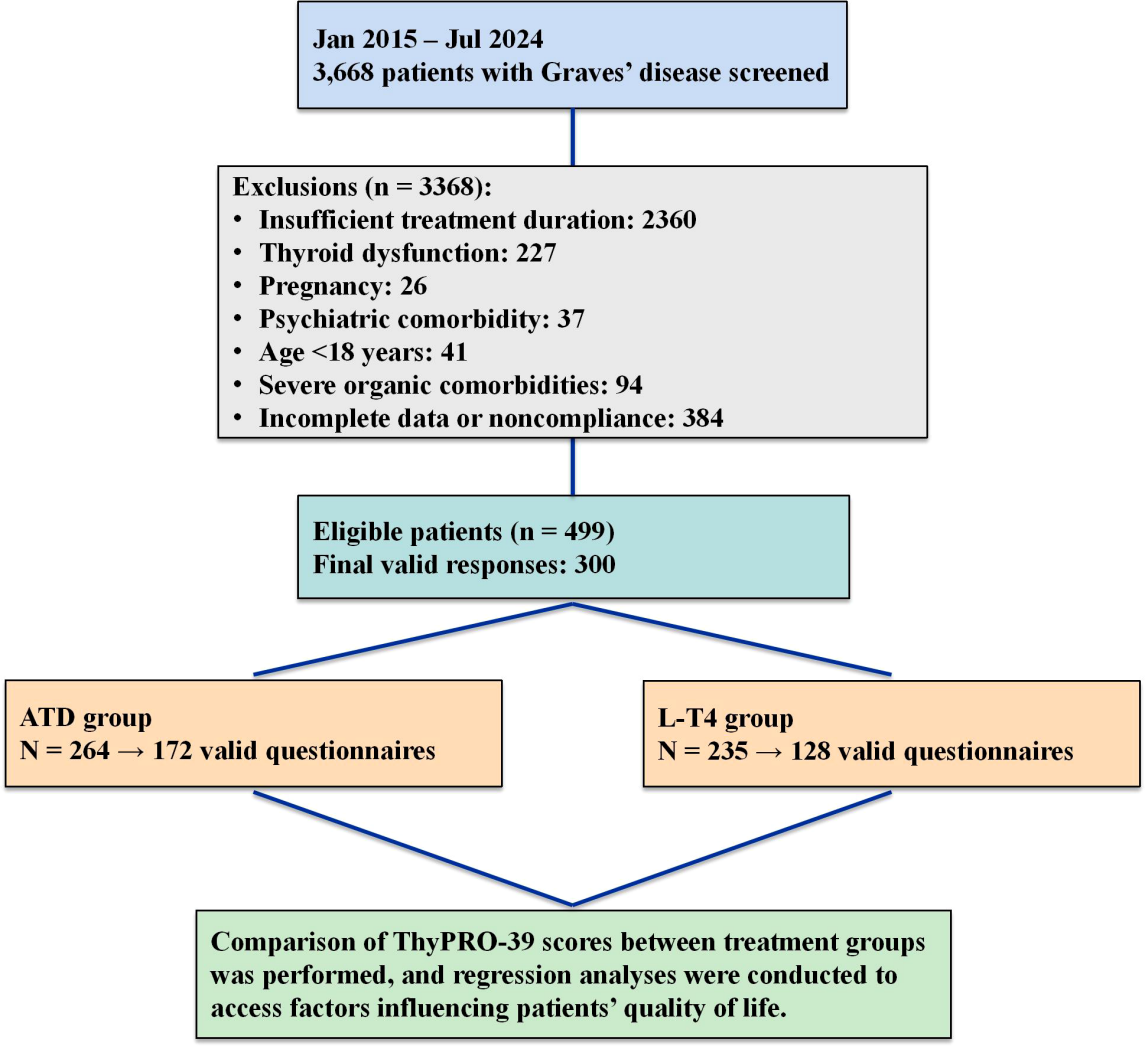
Research process flowchart.

A total of 499 patients with refractory Graves’ hyperthyroidism were ultimately enrolled based on inclusion and exclusion criteria. Among them, 264 patients received long-term ATDs therapy (ATDs group), and 235 received long-term L-T4 replacement following radioactive iodine-131 therapy (L-T4 group) (**Figure 1**). Baseline characteristics and follow-up data for both groups are presented in **Tables 1**, **2**.

**Table 1:** Baseline characteristics of patients in the ATDs group and L-T4 group.

	ATDs group (N = 264)	L-T4 group (N = 235)	*P*-value
Sex, n (%)			0.097
Female	220 (83.3)	182 (77.4)	
Male	44 (16.7)	53 (22.6)	
Age at onset (years)	31 (23–44)	32 (25–43)	0.689
Disease duration (months)	45 (33–66)	51 (37–84)	0.006
Chronic comorbidities, n (%)			0.395
Present	22 (8.3)	26 (11.1)	
Absent	242 (91.7)	209 (88.9)	
Graves’ orbitopathy, n (%)			0.001
Present	66 (25.0)	25 (10.6)	
Absent	198 (75.0)	210 (89.4)	
History of hyperthyroidism relapse, n (%)			0.041
Yes	41 (15.5)	54 (23.0)	
No	223 (84.5)	181 (77.0)	
Duration of treatment (months)	40 (30–55)	35 (26–41)	0.21
TSH (mIU/L)	0.005 (0.005–0.005)	0.005 (0.005–0.005)	0.912
FT3 (pmol/L)	21.24 (13–22.99)	24 (14.86–34.55)	<0.001
FT4 (pmol/L)	51.07 (28.8–62.74)	54.22 (37.7–88.77)	0.001
TRAb (IU/L)	17.52 (9.54–19.41)	17.65 (9.69–22.34)	0.575
TgAb (IU/mL)	54.12 (14.4–284.4)	202.5 (21.1–621)	0.002
TPOAb (IU/mL)	105.3 (14.9–370)	196 (55.6–397.4)	0.036
Baseline thyroid volume (mL)	11.08 (7.82–17.18)	22.07 (14.74–30.57)	<0.001

Reference ranges: TSH, 0.27–4.2 mU/L; FT3, 3.60–7.50 pmol/L; FT4, 12.0–22.0 pmol/L; TRAb, <1.75 IU/L; TgAb, <115 IU/mL; TPOAb, <34 IU/mL.

**Table 2:** Follow-up characteristics of patients in the ATDs and L-T4 groups.

Variables	ATDs group (N = 264)	L-T4 group (N = 235)	*P*-value
Current medication dose			
Methimazole (mg)	2.86 (2.5–6.25)	–	
Levothyroxine (μg)	–	75 (50–100)	
TSH (mU/L)	1.85 (0.63–2.57)	2.03 (0.54–4.83)	0.003
FT3 (pmol/L)	5.02 (4.59–5.38)	4.49 (4.09–4.94)	<0.001
FT4 (pmol/L)	15.9 (14.1–17.5)	19.05 (17–20.85)	<0.001
FT3/FT4 ratio	0.31 (0.27–0.36)	0.23 (0.21–0.27)	<0.001
TSHI	2.48 (0.4–3.08)	3.28 (1.87–4.14)	<0.001
TT4RI	24.53 (2.87–40.18)	35.35 (9.42–92.84)	<0.001
TRAb (IU/L)	2.12 (1.61–5.83)	3.4 (1.73–6.01)	0.36
Thyroid volume at follow-up (ml)	16.49 (11.73–24.51)	–	
Estimated average 2-year treatment cost (RMB)	2610	2320	

Reference ranges: TSH, 0.27–4.2 mU/L; FT3, 3.60–7.50 pmol/L; FT4, 12.0–22.0 pmol/L; TRAb, <1.75 IU/L; TgAb, <115 IU/mL; TPOAb, <34 IU/mL.

In the ATDs group, 220 patients (83.3%) were female. The median age at disease onset was 31 years (interquartile range [IQR], 11). A total of 66 patients (25.0%) had coexisting Graves’ orbitopathy, and 41 (15.5%) had a history of hyperthyroidism relapse. Seven patients had received long-term treatment with propylthiouracil, while the remaining 257 were treated with long-term methimazole (MMI).

In the L-T4 group, 182 patients (77.4%) were female and 53 (22.6%) were male. The median age at onset was 32 years (IQR, 18). Graves’ orbitopathy was present in 25 patients (10.6%), and 54 patients (23.0%) had a prior history of relapse. All patients in this group had received long-term levothyroxine therapy following radioactive iodine treatment.

**Figure 2:**
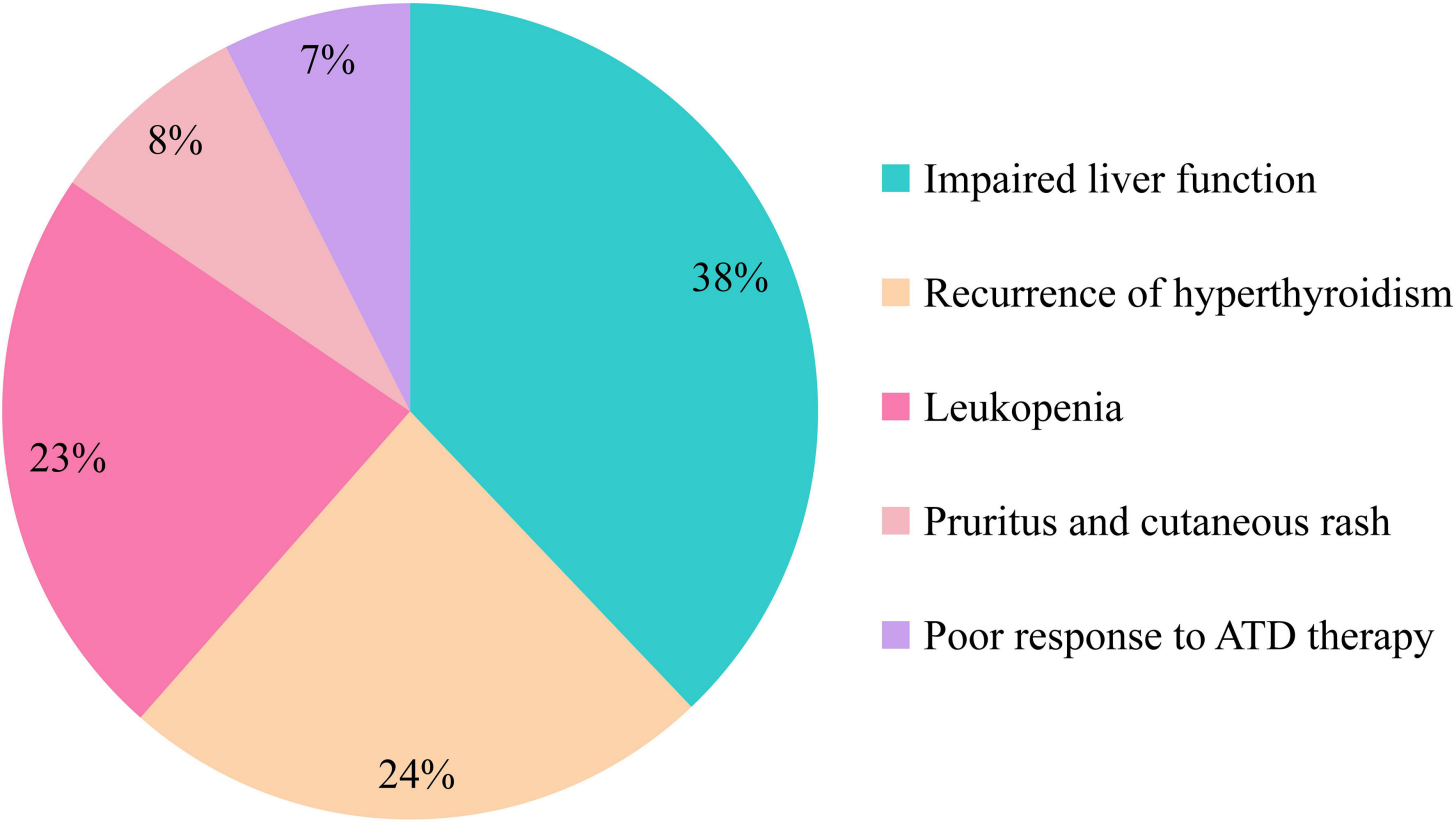
Reasons for choosing iodine-131 therapy among patients in the L-T4 group (n = 211 respondents who provided reasons).

Among the 235 patients in the L-T4 group, 14 received iodine-131 as their initial treatment, whereas 221 were initially managed with ATDs and subsequently switched to iodine-131 therapy. The reasons for selecting iodine-131 therapy in the L-T4 group are summarized in. Of the 211 patients who provided this information, the most common indication was adverse reactions to ATDs. Specifically, 80 patients (38%) experienced drug-induced liver dysfunction, 48 (23%) developed leukopenia, and 17 (8%) presented with allergic skin reactions. In addition, 52 patients (24%) underwent iodine-131 therapy due to recurrent hyperthyroidism, and 14 (7%) received it because of poor therapeutic efficacy of ATDs (**Figure 2**).

At baseline, compared with the ATDs group, patients in the L-T4 group had a significantly longer disease duration (95% confidence interval (CI): 0.004, 0.008; P = 0.006), a lower prevalence of Graves’ orbitopathy (95% CI: 0.001, 0.002; P = 0.001), and a higher proportion with a history of hyperthyroidism relapse (95% CI: 0.036, 0.45; P = 0.041). In terms of thyroid function, the L-T4 group exhibited significantly higher baseline levels of FT3 (P < 0.001), FT4 (95% CI: 0.001, 0.002; P = 0.001), TgAb (95% CI: 0.001, 0.003; P = 0.002), and TPOAb (95% CI: 0.031, 0.040; P = 0.036). No significant differences were observed between the two groups with respect to sex distribution, age at onset, the presence of chronic comorbidities, baseline TSH, or baseline TRAb levels (all P > 0.05).

At follow-up, the L-T4 group showed significantly higher levels of TSH (95% CI: 0.002, 0.004; P = 0.003) and FT4 (P < 0.001), whereas FT3 levels were lower compared with the ATDs group (P < 0.001). Thyroid hormone sensitivity indices demonstrated that patients in the ATDs group exhibited greater sensitivity. The ATDs group had a higher FT3/FT4 ratio (P < 0.001), indicating stronger peripheral sensitivity to thyroid hormones. In addition, the ATDs group showed lower TSHI (P < 0.001) and lower TT4RI (P < 0.001), consistent with better central thyroid hormone sensitivity compared with the L-T4 group. TRAb levels at follow-up did not differ significantly between the two groups (P = 0.36). Additionally, treatment cost estimates were calculated based on the average daily medication dose, the frequency of laboratory and imaging tests, and outpatient registration fees. Under the assumption of relatively stable dosing and follow-up schedules over a 24-month period, the estimated 2-year average cost per patient was slightly higher in the ATDs group (¥2,610) than in the L-T4 group (¥2,320). Treatment cost was reported descriptively due to potential heterogeneity and incomplete capture. Therefore, no formal between-group statistical testing was performed.

### 3.2 QoL outcomes

**Figure 3:**
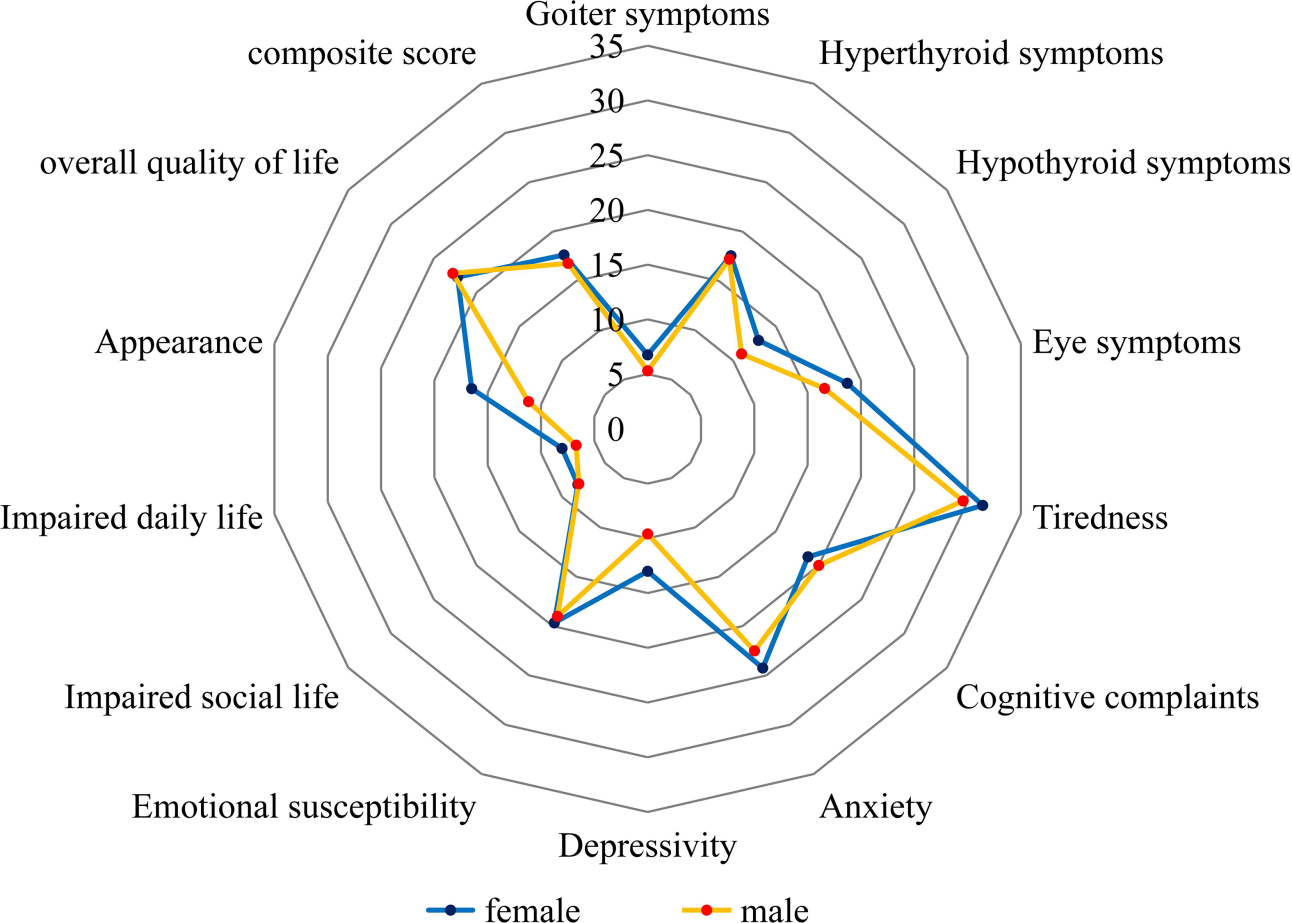
Radar chart comparing mean ThyPRO-39 scores between patients in the ATD group and the L-T4 group.

A total of 300 patients completed the ThyPRO-39 questionnaire, including 172 in the ATDs group and 128 in the L-T4 group (**Figure 3**). A comparison of the subscale and composite scale scores between the two groups is presented in **Table 3**. The results showed that patients in the L-T4 group had significantly higher scores in the following subscales: hyperthyroid symptoms, hypothyroid symptoms, eye symptoms, anxiety, emotional susceptibility, impaired social life, impaired daily life, appearance, overall QoL, as well as in the composite score (P < 0.05). These findings indicate that patients in the L-T4 group experienced poorer QoL compared to those in the ATDs group. Regarding the additional study-specific item, “After taking your medication, do you feel that your QoL has partially or completely returned to its pre-morbid level?”, 158 patients in the ATD group (91.8%) reported a perceived recovery of 50% or greater, while 106 patients in the L-T4 group (82.8%) reported the same.

**Table 3:** Comparative ThyPRO-39 scores in patients receiving ATD therapy versus L-T4 therapy.

Variables	ATD group (N = 172)	L-T4 group (N = 128)	*P*-value
Sex, n (%)			
Female	146 (84.9)	101 (78.9)	
Male	26 (15.1)	27 (21.1)	
Goiter symptoms	0 (0–16.67)	0 (0–8.33)	0.116
Hyperthyroid symptoms	12.5 (6.25–18.5)	18.75 (12.5–31.25)	0.005
Hypothyroid symptoms	12.5 (6.25–18.75)	18.75 (7.81–18.75)	<0.001
Eye symptoms	16.67 (8.33–25)	16.67 (8.33–33.33)	0.004
Tiredness	33.33 (25–33.33)	33.33 (25–41.67)	0.775
Cognitive complaints	16.67 (8.33–25)	16.67 (8.33–33.33)	0.087
Anxiety	16.67 (8.33–25)	20.05 (8.33–33.33)	0.019
Depressivity	16.67 (8.33–16.67)	16.67 (8.33–16.67)	0.542
Emotional susceptibility	16.67 (8.33–25)	25 (16.67–33.33)	<0.001
Impaired social life	0 (0–16.67)	8.3 (0–16.67)	0.006
Impaired daily life	8.33 (0–16.67)	8.33 (0–16.67)	0.004
Appearance	8.33 (8.33–16.67)	16.67 (8.33–25)	0.020
Overall quality of life	25 (0–25)	25 (0–50)	0.026
Composite score	15.91 (11.36–20.45)	20.45 (14.77–21.59)	<0.001
QoL improvement ≥50%, n (%)	158 (91.8)	106 (82.8)	0.454

**Figure 4:**
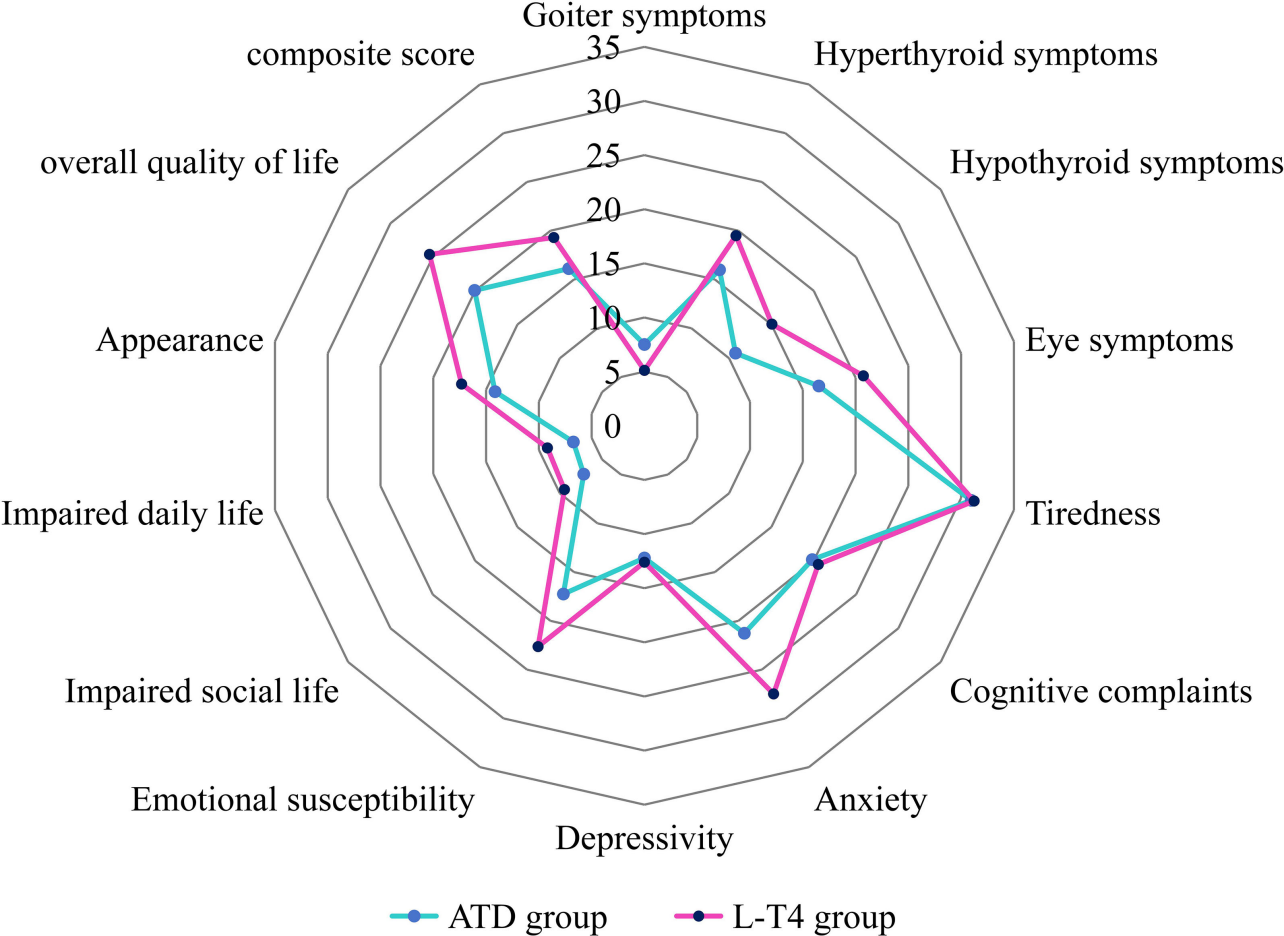
Radar chart comparing mean ThyPRO-39 scores between male and female patients.

Patients who completed the ThyPRO-39 were also grouped by sex, with 247 females and 53 males (**Figure 4**). As shown in **Table 4**, female patients had significantly higher scores than males in the depression and appearance subscales (P < 0.05), suggesting that women may be more prone to depressive symptoms and concerns related to appearance.

**Table 4:** Comparison of ThyPRO-39 scores between male and female patients.

Variables	Female (N = 247)	Male (N = 53)	*P*-value
Goiter symptoms	0 (0–16.67)	0 (0–8.33)	0.391
Hyperthyroid symptoms	12.5 (6.25–31.25)	12.5 (12.5–18.75)	0.903
Hypothyroid symptoms	12.5 (6.25–18.75)	12.5 (6.25–18.75)	0.116
Eye symptoms	16.67 (8.33–25)	16.67 (8.33–25)	0.248
Tiredness	33.33 (25–41.67)	28.12 (25–33.33)	0.175
Cognitive complaints	16.67 (8.33–25)	18.61 (8.33–25)	0.627
Anxiety	25 (8.33–33.33)	25 (8.33–33.33)	0.472
Depressivity	16.67 (8.33–16.67)	8.33 (0–16.67)	0.005
Emotional susceptibility	16.67 (10.42–25)	16.67 (8.33–31.25)	0.583
Impaired social life	0 (0–16.67)	8.3 (0–16.67)	0.720
Impaired daily life	8.33 (0–16.67)	8.33 (0–8.33)	0.331
Appearance	16.67 (8.33–22.92)	8.33 (0–16.67)	0.003
Overall quality of life	25 (0–25)	25 (0–25)	0.722
Composite score	18.18 (13.64–21.59)	15.91 (12.5–21.31)	0.189
QoL improvement ≥50%, n (%)	219 (88.7)	45 (84.9)	0.154

### 3.3 Evaluate the influencing factors of QoL

Multivariate linear regression analyses were conducted using each of the ThyPRO-39 subscale scores and the composite score as dependent variables. Independent variables included sex, disease duration, presence of Graves’ orbitopathy, history of hyperthyroidism relapse, baseline FT4, baseline TPOAb, baseline thyroid volume, follow-up TSH, follow-up FT4, presence of chronic comorbidities, and treatment group. Categorical variables, including sex, treatment group, presence of Graves’ orbitopathy, and history of relapse, were coded as binary variables (0 or 1) as follows: ATD group = 0 (reference) and L-T4 group = 1; female = 0 (reference) and male = 1; no chronic comorbidities = 0 (reference) and presence of chronic comorbidities = 1; no Graves’ orbitopathy = 0 (reference) and presence of Graves’ orbitopathy = 1; no history of hyperthyroidism relapse = 0 (reference) and history of hyperthyroidism relapse = 1. Collinearity diagnostics showed that all variables had tolerance values ranging from 0.478 to 0.929 (all > 0.1), and variance inflation factors (VIFs) ranging from 1.077 to 2.091 (all < 10), indicating no significant multicollinearity among the independent variables; therefore, no variables were excluded from the models.

The independent variables that have a statistically significant impact on the dependent variable in each model are summarized (**Table 5**). The multiple linear regression analysis shows that female patients have a positive correlation with the scores of the three subscales of hypothyroidism symptoms (P = 0.018), tiredness (P = 0.046), and depressivity (P = 0.014). This suggests that female patients may be more likely to experience fatigue, depression, and related hypothyroid symptoms than male patients. The history of hyperthyroidism recurrence is positively correlated with the scores of the two subscales of eye symptoms (P = 0.031) and anxiety (P < 0.001). Female patients and patients in the L-T4 group were both positively associated with higher composite scale scores (P = 0.023 and P = 0.046, respectively). These findings suggest that female patients and those undergoing long-term L-T4 replacement therapy after radioiodine treatment may be associated with poorer QoL.

**Table 5:** Results of multiple linear regression analysis.

Dependent variable	Independent variable	B	95% CI	Standardized β	R²	Adjusted R²	*P* value	Tolerance	VIF
Hypothyroid symptoms	Sex	-4.793	-6.835, 0.078	-0.226	0.311	0.248	0.018	0.827	1.210
Eye symptoms	Graves’ ophthalmopathy	11.329	6.671, 15.525	0.410	0.193	0.137	<0.001	0.880	1.136
	Recurrence of hyperthyroidism	6.453	2.835, 10.560	0.214			0.031	0.654	1.529
Tiredness	Sex	-5.100	-7.324, 0.035	-0.195	0.296	0.219	0.046	0.827	1.210
Anxiety	Recurrence of hyperthyroidism	19.461	17.184, 29.457	0.459	0.332	0.285	<0.001	0.654	1.529
Depression	Sex	-5.096	-7.209, 0.009	-0.237	0.213	0.196	0.014	0.827	1.210
Overall QoL	Baseline FT4	-0.166	-0.206, 0.660	-0.204	0.347	0.324	0.027	0.870	1.149
	TSH at follow-up	1.200	0.015, 1.904	0.280			0.005	0.745	1.342
Composite score	Treatment group	2.462	1.285, 5.355	0.243	0.367	0.286	0.046	0.478	2.091
	Sex	-2.811	-3.858, 0.649	-0.211			0.023	0.827	1.210

## 4 Discussion

In this single-center cross-sectional study, patients in the L-T4 group were associated with poorer QoL compared to those receiving long-term ATDs treatment. Given the cross-sectional design, the observed differences in ThyPRO-39 scores should be interpreted as associations rather than causal effects of treatment. The conclusion of this study is in line with some previous studies. A 2019 multicenter, long-term follow-up study comparing three treatment strategies found that patients treated with radioactive iodine (RAI) fared significantly worse than those receiving ATDs or surgery on both thyroid-specific and generic QoL measures, with particularly pronounced deficits in anxiety, depression, social functioning, goiter-related symptoms and hyperthyroid symptoms (24). Concordant evidence comes from a 10-year retrospective analysis by Azizi et al., which showed superior QoL in patients maintained on prolonged ATDs therapy versus those managed with RAI (15). Likewise, a cohort followed for an average of 14 years demonstrated that methimazole-treated patients outperformed their RAI-treated patients in mood, executive function and memory domains (25). A 25-year Norwegian study of 182 individuals further reported poorer QoL at follow-up among patients who developed hypothyroidism after RAI than among those who remained on ATDs (26, 27). On the contrary, some studies have found that there is no significant difference in the QoL among patients with Graves’ disease who receive different treatment methods (18, 28, 29). However, these studies have small sample sizes and did not use thyroid disease-specific scales to assess the QoL, lacking certain sensitivity. Moreover, compared with limited-scale randomized studies, patient-centered open research designs may better reflect the real results.

To elucidate factors associated with patients’ QoL, we conducted a multiple linear regression analysis. The results showed that female patients and those who received long-term L-T4 replacement therapy after radioactive iodine-131 treatment were associated with poorer QoL. Moreover, female patients were more likely to experience fatigue, depression, concerns about appearance, and related symptoms of hypothyroidism compared to male patients. Like most autoimmune diseases, GD is more prevalent in females than in males. This gender difference in prevalence also exists in some long-term complications of GD, such as depression and anxiety (26). In the general population, the prevalence of depression and anxiety is inherently higher among women than men (30). A long-term follow-up study from Sweden, involving 1,186 patients with GD, reported findings like our study (31). Despite successful control of hyperthyroidism and maintenance of euthyroidism, female patients were more likely to experience long-term issues such as depression, impaired sexual function, and body image concerns, all of which negatively affected their QoL (31). Similarly, a cross-sectional study from Brazil, which included 154 patients with GD and used the SF-36 questionnaire to assess QoL, found that female sex and the presence of Graves’ orbitopathy were significantly associated with poorer QoL (32). These studies collectively suggest that female patients with GD may be more prone to experiencing reduced QoL. However, it remains unclear whether this reduced QoL is primarily attributable to the disease itself or reflects the generally higher baseline prevalence of these symptoms among women in the broader population. Further research is needed to determine this.

Previous studies have reported that visual impairment and psychological distress due to changes in appearance are common among patients with GD complicated by orbitopathy (33–35). RAI therapy has been shown to increase the risk of developing or exacerbating thyroid eye disease, which may be related to poorer QoL in patients undergoing RAI. In our study, the majority of patients in the L-T4 group had previously received ATDs therapy prior to RAI. Transition to RAI was often due to adverse reactions to ATDs, such as hepatotoxicity, leukopenia, and dermatological reactions, or due to inadequate therapeutic response or recurrence of hyperthyroidism during treatment. These factors suggest that patients in the L-T4 group may have had a longer disease duration and more complex clinical course, which could partially explain their poorer QoL compared to patients treated with ATDs alone. Furthermore, some studies have suggested that RAI therapy may trigger increased exposure of thyroid antigens, leading to dysregulation of the autoimmune response and a temporary rise in TRAb levels following treatment (36). Whether TRAb or other thyroid-specific autoimmune markers exert long-term negative effects on the QoL in patients with GD remains uncertain and warrants further investigation.

Even when thyroid function remains stable within the normal range under long-term L-T4 replacement therapy, patients’ QoL may still be affected. Studies have shown that during the period of thyroid hormone replacement therapy for patients with hypothyroidism, even if their thyroid function remains within the normal range, their mental health status is poorer compared to those without thyroid disorders (37). A study from the United Kingdom involving 697 patients on thyroid hormone replacement therapy found that variations in FT4 and TSH levels, even when within reference ranges, could significantly impact psychological health in hypothyroid patients (38). In addition, thyroid hormone sensitivity may also influence QoL. Previous studies have suggested that in euthyroid individuals, a low FT3/FT4 ratio is increasingly recognized as being closely associated with depression, greater fatigue, and reduced QoL (39). Our findings show that patients in the ATDs group exhibited higher peripheral and central sensitivity to thyroid hormones compared with those in the L-T4 group. One explanation is that radioiodine therapy may lead to reduced deiodinase activity, particularly type 2 deiodinase (DIO2), which is crucial for local conversion of T4 to T3 in brain and peripheral tissues. Patients on L-T4 monotherapy following radioiodine or thyroidectomy report lower free T3 levels even when TSH and FT4 are within normal ranges, consistent with impaired local T4→T3 conversion impacting clinical symptoms and wellbeing (40, 41). Another aspect is the distinction between exogenously supplied T4 and endogenously produced T3. While exogenous T4 is converted peripherally to T3, endogenously produced T3 (from thyroid tissue) may more directly modulate TSH suppression and preserve normal thyroid hormone action at target tissues. Loss of intrinsic thyroid function caused by ablation, radioiodine, or suppression of thyroid tissue could therefore reduce the generation of endogenous T3 (24, 40, 42, 43).

Although the main treatment options for Graves’ hyperthyroidism have remained largely unchanged over the past several decades, the management of the condition remains complex. In making clinical decisions, physicians should not only consider the disease itself, the patient’s age, comorbidities, and pregnancy plans, but also take into account individual treatment preferences. Furthermore, during disease monitoring and treatment evaluation, it is important to pay attention not only to objective clinical indicators but also to patients’ subjective experiences, in order to optimize both disease control and QoL (44, 45). This is a single-center study conducted in a tertiary academic referral center, which may limit generalizability. Results should not be directly extrapolated to other institutions or healthcare systems without external validation.

This study has several limitations (1): It lacks baseline quality of life data, making it impossible to evaluate patients’ quality of life prior to treatment, meaning that we cannot determine whether observed differences reflect pre-existing group differences rather than treatment-related differences (2). Of 499 eligible patients, only 300 completed the ThyPRO-39 questionnaire (response rate 60.1%), which may introduce non-response bias, potentially affecting the validity and generalizability of our findings (3). Socioeconomic factors such as income level, educational background, and marital or reproductive status were not included in the analysis, which may limit the comprehensiveness of the conclusions (4). This study was based on retrospective data collection and selection, which may introduce certain selection bias (5). Treatment allocation was not randomized. Radioiodine was commonly chosen due to relapse or adverse reactions to ATDs, introducing confounding by indication. Although multivariable adjustment was performed, residual confounding from unmeasured factors may persist (6). Excluding patients with diagnosed psychiatric disorders may have underestimated QoL impairment and limited external validity, potentially biasing our results toward more favorable QoL estimates in an unselected Graves’ disease population (7). The subscale analyses involve multiple testing, which may increase the risk of Type I error (false positives).

## 5 Conclusion

This study utilized the thyroid disease-specific ThyPRO-39 questionnaire to compare the QoL in patients with refractory Graves’ hyperthyroidism who underwent either long-term ATDs therapy or long-term L-T4 replacement therapy following iodine-131 treatment. Additionally, regression analysis was employed to explore the factors associated with QoL. The results indicated that both gender and treatment modality were significant factors associated with QoL in patients with GD. Compared to those receiving long-term ATDs therapy, patients undergoing long-term L-T4 replacement after RAI treatment were associated with poorer QoL. Moreover, female patients were also associated with poorer QoL than male patients, and were more likely to experience fatigue, depression, body image concerns, and symptoms related to hypothyroidism. In view of the cross-sectional design and potential residual confounding, it is recommended that these findings be interpreted as associations rather than as evidence of treatment effects. Prospective, multicenter studies with baseline QoL assessment are warranted.

## Data Availability

The raw data supporting the conclusions of this article will be made available by the authors, without undue reservation.
